# Association between Health Indicators and Health-Related Quality of Life according to Physical Activity of Older Women

**DOI:** 10.3390/healthcare8040507

**Published:** 2020-11-22

**Authors:** Hee-Sook Lim, Jeong-Ju Yoo

**Affiliations:** 1 Department of Food and Nutrition, Yeonsung University, Anyang 14011, Korea; limhs@yeonsung.ac.kr; 2 Division of Gastroenterology and Hepatology, Department of Internal Medicine, Soonchunhyang University Bucheon Hospital, Bucheon 14854, Korea

**Keywords:** health services for the aged, exercise, health care, health-related quality of life, diet

## Abstract

The purpose of this study was to compare sociodemographic factors, health factors and nutritional status according to the physical activity of older women, and to analyze the factors affecting their quality of life. The subjects of this study were 5661 older women aged 60 or older who participated in the Korean National Health and Nutrition Survey (KNHANES) from 2008 to 2013. The socioeconomic factors, subjective health status and disease status, lifestyle and dietary quality, and life quality were compared among two groups (active group and inactive group). The inactive group had significantly higher rates of obesity and unemployment, comorbidities, numbers without spouses, experiencing stress, poor subjective health status, depression or suicidal thoughts, and also higher rates of skipping meals. The life quality index and dietary quality was also significantly lower in the inactive group, and subjects experienced significantly higher pain or discomfort and problems in mobility and the usual activities. The results of multivariate analysis after adjusting for age in groups engaging in physical activities showed the life quality index to increase in accordance with the diet quality, economic income, and education level. It was confirmed that life quality was significantly low if the participant showed a poor subjective health evaluation, obese with many diseases, spouseless, and experienced high levels of stress. Considering the rapid aging and high life expectancy of women, regular physical activity is very important for maintaining health and improving the life quality of older women, and it is believed that comprehensive attention and management of lifestyle and diet quality are necessary.

## 1. Introduction

Due to the rapid aging of Korea, the demand for health care and medical expenditure are increasing [1]. In order to promote the health of older adults, various goals have been established for the development and application of many programs [2]. Health problems of the elderly can be prevented or delayed mainly through changes in health behavior and lifestyle, and chronic diseases of older adults are often approached in a preventive way to improve the quality of health-related quality of life (HRQoL) through management and maintenance rather than solely reliance on cures and treatments [3,4]. HRQoL can be defined as “how well a person functions in their life and his or her perceived wellbeing in physical, mental, and social domains of health” [5]. Therefore, HRQoL is judged to have a different meaning from the quality of life or health.

Recently, much research has been conducted on enhancing physical function or preventing muscle loss [6,7]. The increase in body fat and sarcopenia in older adults is known to increase the risk of metabolic diseases and fractures, whereas physical activities can help reduce the risk of chronic diseases such as hypertension, diabetes, and stroke [8,9,10,11]. It is reported that physical activities reduce the risk of diseases and help prevent depression and fractures [12]. In addition, activity improves physical strength and prevents physiological function decline, thereby increasing the independence of older adults, ultimately improving their quality of life [11,13,14].

The World Health Organization (WHO) recommendation for the physical activity of the older adults aged 65 years and above is at least 150 min of moderate-intensity aerobic physical activity or 75 min or more of high-intensity aerobic physical activity or strength-enhancing physical activity per week [15]. Also, for older adults with poor mobility, the WHO recommends 3 days or more per week of physical activity to enhance balance and prevent falls. However, the physical activity practice rate of the older adults in Korea is low, not even reaching 50% of the target [16]. In addition, Koreans have exceeded the Organization for Economic Cooperation and Development (OECD) average life expectancy, and the life expectancy of Korean females is 85.4 years [17]. Also, the prevalence of chronic diseases in older women is higher than males. When considering previous studies that show physical activities of older women differing greatly by region and economic conditions [18], it is necessary to provide more active and diverse physical activity programs. However, multifarious domestic studies on the physical activities of older women are still insufficient. This study conducted a comprehensive investigation on the differences in health status, nutritional status, and life quality according to regular physical activities of older women. The results of this study will contribute as basic data for the development of social service programs.

## 2. Materials and Methods

### 2.1. Study Subjects

**Figure 1:**
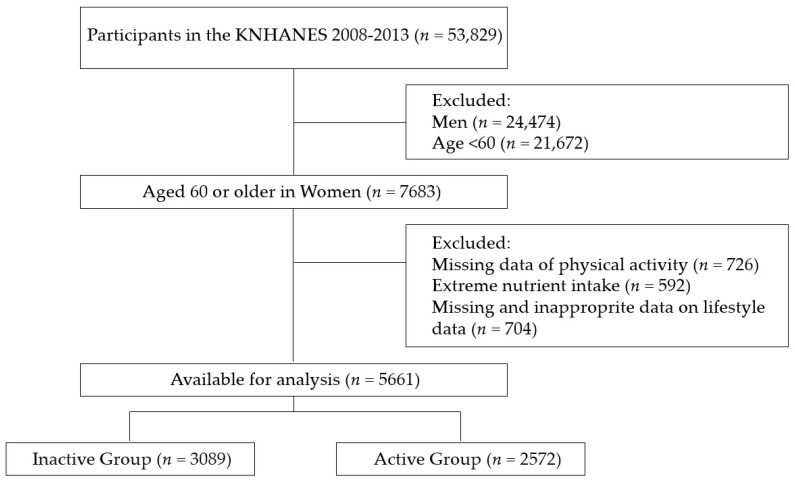
Study population.

This study used data from the Korea National Health and Nutrition Examination Survey (KNHANES) conducted from 2008 to 2013. Among the total of 53,829 participants, any exclusion criteria—such as data of men, women under 60 years, or missing data regarding physical activity and lifestyle—were excluded from the analysis. The final participants totaled 5661 and they were organized into 2 different categories named “Inactive Group (*n* = 3089)” and “Active Group (*n* = 2572)” depending on the level of their physical activity (Figure 1). Among the total participants, healthy older adults were 898 (15.9%) and adults with more than one chronic disease numbered 4763 (84.1%). The survey and data were reviewed by the Institutional Review Board of the Korea Centers for Disease Control and Prevention and were approved by the Ethics Committee of the Korea Centers for Disease Control and Prevention (2007-02CON-04-P, 2008-04EXP-01-C, 2009-01COM-03-2C, 2010-02CON-21-C, 2011-02CON-06-C, 2012-01EXP-01-2C, 2013-07CON-03-4C, and 2013-12EXP-03-5C).

The international physical activity questionnaire–short form (IPAQ-SF) was used to estimate the overall physical activity (PA) level of an individual in metabolic equivalent (MET)-min/week by determining the duration (in minutes) and number of days (in 1 week) of engagement in three specific types of activity (walking, moderate-intensity activities, and high-intensity activities) across a comprehensive set of domains (leisure time, work-related and transport-related physical activities, and domestic and gardening activities) during the past 7 days [19]. The IPAQ-SF has shown acceptable reliability and validity [20,21] in previous studies. According to the international physical activity evaluation scoring system based on WHO Physical Activity Guidelines [22] those who performed medium and high-intensity physical activity (medium to vigorous physical activity, MVPA) for a total of 150 min or more per week were classified as ‘Active group’, and those who were engaged in ‘Inactive group’. The time spent on MVPA was calculated as the sum of the time spent on medium- and high-intensity activities according to the IPAQ scoring protocol [23].

### 2.2. Variables

#### 2.2.1. General Factors

For the general factor, the age and body mass index were calculated, and the participants were classified into underweight, normal, overweight and obese [24]. For the income level, the participants were classified into low, mid-low, mid-high, and high incomes depending on the amount of their monthly incomes. For the educational factor, participants were classified into middle school, high school, and college graduates. Regarding occupation, participants were classified into blue-collar workers (service and sales workers, agriculture, forestry and fishery-related elementary workers, machine operation and controller, and other service-related elementary workers), white-collar workers (managers, experts, and office workers) and non-workers. Additional factors such as the existence of national basic living allowance, current spouse status, and residential area were also included [25].

#### 2.2.2. Health Conditions

In terms of subjective health status and body image, general questionnaires were used to gather data from participants. Regarding diseases, participants were considered to have a disease if the participants were diagnosed by doctors as having problems in the respiratory system, endocrine system, musculoskeletal system, or with depression, chronic renal failure, cancer, or hepatitis. Depending on the number of diseases, the result was categorized into “no medical history” as (0) or “existing medical history” as (1) [25].

#### 2.2.3. Lifestyle and Life Quality

The sleep time of participants was determined through questionnaires, with the average recommended sleep time of 8 h per day as its base standard. Moreover, stress perception, level of depression, current smoking status, and regular meals per day were also analyzed. Health-related quality of life was developed EQ-5D by the EuroQoL Group to measure overall health. In Korea, it has been modified and used in the 3rd National Health and Nutrition Survey. The EQ-5D consists of five dimensions (mobility, usual activities, self-care, pain or discomfort, and anxiety or depression) with three response levels for each dimension (no problem, moderate problem, and severe problem) generating a different health state [26,27]. EQ-5D is represented by a single score between 0 and 1 by weight.

#### 2.2.4. Nutritional Status

The nutrition survey used the face-to-face interview method and the eating habits and food intake questionnaire has been designed as an open-ended survey for reporting various dishes and foods using the 24 h-recall method with various measuring aids [25]. Nutrients intake was analyzed by daily energy, protein, fat, sugar, fiber, calcium, phosphorus, iron, sodium, potassium, vitamin-A, retinol, carotene, vitamin B_1_, vitamin B_2_, niacin, and vitamin C. The quality of diet was calculated based on Nutrient Adequacy Ratio (NAR) and the Mean Adequacy Ratio (MAR). The NAR calculated from the recommended nutrient intake represents the gender-specific and age-specific requirements of daily intake. A NAR value close to 1 indicates that the amount of nutrient intake is close to the daily recommended intake [28,29]. A value higher than 1 suggests that the intake exceeds the daily recommended intake, while a value less than 1 implies that the daily intake is not adequate to meet the daily recommended intake [29].

### 2.3. Statistical Analyses

The clinical and nutrient-related factors of the subjects were summarized as means and standard deviations for continuous variables, and frequency and percentage (%) for categorical variables. The significant differences between the active and inactive groups were identified by an independent 2-sample *t*-test and Wilcoxon’s rank-sum test for continuous data and Fisher’s exact test for categorical data. Linear regression models were also carried out to access the quality of life in older women. Results are expressed as b coefficients and 95% confidence intervals (CIs). All the analyses were conducted using R (version 3.6.1; The R Foundation for Statistical Computing, Vienna, Austria) and the statistical significance was set at 0.05 based on the 2-sided test.

## 3. Results

### 3.1. Demographic Status according to Physical Activity

The comparison results between the two groups (inactive and active) regarding general and socio-economic factors are shown in Table 1. The average age for “Active Group” was 68.4 and the average age for “Inactive Group” was 69.7 (26% of the inactive group were over 75). The average body mass index did not show a significant difference between the two groups, but the inactive group’s ratio of under-weight and over-weight were significantly higher than that of the active group. In terms of jobs, 38% of the active group was currently employed compared to 30.9% employment of the inactive group. Regarding marital status, 78.6% of the active group and 67.1% of the inactive group had husbands.

### 3.2. Health Conditions and Disease Status according to Physical Activity

The comparison results between the two groups (inactive and active) depending on their health conditions and disease status are shown in Table 2, respectively. The self-assessment questionnaire regarding current subjective health status showed the inactive group’s health condition responses to be significantly worse than that of the active group. Regarding the subjective body type questionnaire, although the obesity rate of the inactive group was higher than that of the active group, more participants from the inactive group believed they had normal or under-weight body types compared to active group participants. Regarding disease status, the comorbidity rate was 83% for the active group and 85.1% for the inactive group. The ratio of having three or more diseases was 18% for the active group and 20.3% for the inactive group.

### 3.3. Lifestyle and Quality of Life according to Physical Activity

The results for lifestyle and quality of life are shown in Table 3. Regarding stress level perception, 6% of the inactive group felt extremely stressed and 23.1% somewhat stressed while only 4.5% of the active group felt extremely stressed and 20.6% somewhat stressed. Moreover, the inactive group showed a much higher depression rate with 24.5% experiencing suicidal thoughts while only 17.9% of the active group had thoughts about committing suicide. In terms of regularity of meals, the inactive group tended to skip meals much more often than the active group. The average quality of life index was 0.88 ± 0.37 points in the active group and 0.84 ± 0.19 points in the inactive group. However, the problem rates on mobility, usual activities, and pain or discomfort were much higher in the inactive group than in the active group.

### 3.4. Nutritional Status according to Physical Activity

The results of nutrient intake and dietary quality of the active and inactive groups are shown in Table 4 and Table 5. The average calorie intake showed minimal difference as the active group consumed an average of 1587.03 ± 516.37 kcal per day and the inactive group consumed an average of 1565.20 ± 501.93 kcal per day. However, the active group consumed significantly more fiber, calcium, vitamin A, carotene, and vitamin C than the inactive group, whereas the inactive group consumed significantly more sodium, phosphorous, and niacin than the active group. According to NAR, if the nutrition consumption score is less than the recommended score of 0.75, it is evaluated as insufficient nutrition consumption. The active group showed insufficiency in calcium consumption and the inactive group showed insufficiency in calcium and potassium consumption. For MAR, the active group had a score of 1.10 ± 0.74 and the inactive group had a score of 1.04 ± 0.58. Even though both groups maintained a score of above 1, the active group showed a significantly higher score in MAR.

### 3.5. Factors Affecting the Quality of Life for Older Women

Table 6 shows the results of the analysis of the factors that affect life quality. As a result of performing multiple linear regression analysis after adjusting the age using the variable that showed significant results as a result of single regression analysis, in the case of physical activity (*p* < 0.021), the higher the diet quality (*p* < 0.001), the higher the income and higher education level (*p* < 0.001), the higher the quality of life index. On the other hand, a low score on subjective health status, obesity, diseases, being spouseless, and high levels of stress often led to a lower quality of life index score.

## 4. Discussion

This study used Korean nationwide data to examine health indicators and analyze factors that affect the quality of life for older women over 60 according to their level of physical activity.

Aging causes a muscle-reducing condition called sarcopenia and can cause dysfunction in everyday lives [30]. Therefore, regular exercise is recommended to increase daily physical activity [31]. According to previous studies, regular exercise and intense exercise increase basal metabolism which has a positive effect on the prevention of muscular dystrophy [32,33]. Aerobic exercise causes significant changes in abdominal fat percentage, body fat percentage, and body fat mass [34,35]. In this study, the active group performing regular exercise had a slightly lower obesity rate than the inactive group. Moreover, the employment rate was higher, and the percentage of those with spouses was significantly higher for the active group. Regarding the subjective health condition evaluation, the active group had a higher response rate of being in good health, and the disease history/the current number of diseases was significantly lower than those of the inactive group. Prior studies have also shown that older people who are relatively healthier than others exercise more often than ordinary people, and participate in a wide range of leisure activities [36,37]. Of course, by contrast, one cannot ignore the fact that people with many illnesses inevitably fail to actively participate in any type of physical activity because of physical restrictions and limitations due to the presence and severity of a disease. According to Moon et al., higher income and education level led to a higher level of physical activity, but this study did not show a significant difference [38].

Regarding lifestyle, the active group was less likely to receive stress and think of committing suicide compared to the inactive group. Moreover, the active group was more likely to have a regular eating habit than the inactive group. Kanamori et al. reported that even 10 min of exercise before sleep had a positive effect on blood vessels and reducing depression [39]. In another study, implementing a 48-week exercise program on older adults over 65 with depression reduced the level of depression [40]. As reported in many studies, physical activity can be a very effective intervention strategy for improving depression and stress conditions [41,42,43]. Therefore, professionally developed body management methods that can strengthen the attitudes and motivations toward exercising with a goal to control emotions would be beneficial. As a result of the life quality indicators, the inactive group experienced significantly more problems in athletic mobility, usual activities, and pain or discomfort than the active group. According to Dauwan et al., degraded exercise ability or daily activities can cause diseases that lead to lower quality of life [44]. As a lack of exercise can lead to illnesses, exercising regularly should be emphasized to improve the quality of life and maintain a healthy life.

The results of nutrient status and dietary quality analysis showed that despite no significant difference in the amount of energy and macronutrient, the active group had much greater consumption of vitamins and minerals compared to the inactive group. From the result, it can be deduced that a high level of obesity and skipping meal habits shown in the inactive group correlates with the result. There was also a previous study showing that older women had a higher skipping meals rate and insufficient intake of animal protein, calcium, and folic acid [45]. Despite the majority of women preparing meals, the diversity of dietary intake was lower in women [46]. Nutrients related to body functions are mainly energy, protein, calcium, vitamin D, and vitamin B groups. In particular, vitamin D receptors are an important key to preventing osteoporosis and sarcopenia in the elderly with muscle atrophy [47]. Vitamin D supplementation improved quality of life and physical performance in the elderly [48]. But this study does not have a comparison as there is not enough intervention research on exercise and consumption of older adults in Korea. Since this is a retrospective study, it is only compared to whether or not there is a difference in nutrient intake according to physical activity, but it cannot be interpreted as a causal relationship. However, considering the fact that people who regularly exercise tend to maintain healthy eating habits and consume sufficient nutrients, elderly people should also manage their dietary habits and overall eating amounts. In fact, considering a study that the participation rate and educational effect of regular and repetitive nutrition education is higher in women than in men [49], it is necessary to improve nutritional status by applying customized education for elderly women.

Factors affecting the life quality of older women have been shown to be proportional to physical activities, the quality of diet, household income and education level. On the other hand, the life quality of older women is inversely proportional to their obesity, number of diseases, stress level, and the absence of spouses. The prevalence of hypertension, diabetes, and hypercholesterolemia in Korean older women were significantly higher than men after the age of 60, and the rate of undernutrition was reported to be higher than that of men [50,51]. The WHO has asserted that individuals are significantly affected by their own goals, expectations, standards, and interests in their culture or value system [42]. However, it is difficult to improve the quality of life for elderly people because many other factors than those stated above should also be considered such as body condition, psychological condition, society, and economic condition. Moreover, in the comprehensive evaluation of physical activity/daily living ability, even though men typically were healthier than women, women were more likely to be exposed to depression. The average life expectancy is higher for women than for men, and women are more likely face the psychological burden of living alone, which leads to a lower quality of life. Thus, considering that many more factors can determine and reduce the life quality of women, it is pivotal to explore ways to improve the quality of life for older women.

This study has the advantage of utilizing a large number of data of older women by region. However, retrospective research may contain recall bias and there may be a limitation in explaining causal relationships. It cannot be ruled out that an individual’s non-activity does not depend on health conditions, and the two factors can confuse the causal nature of the phenomena that are explained in correlation.

## 5. Conclusions

The result of analyzing various indicators clearly showed the active group performing regular exercise with a lower obesity rate, less psychological stress and depression compared to the inactive group. Social factors, environmental factors, physical activity, and sufficient nutrient intake were the factors that positively influenced the life quality of older women. Considering that many of these factors can be regulated, it is important to put efforts into providing professional education and management programs to help older women achieve regular exercise and balanced nutrient consumption with the goal of disease prevention and health maintenance.

## Figures and Tables

**Table 1:** General characteristics according to physical activity.

Variables	Active Group	Inactive Group	*p*-Value
	(*n* = 2572)	(*n* = 3089)	
Age	68.4 ± 6.5	69.7 ± 7.1	<0.001
<75	2104 (81.8)	2285 (74.0)	<0.001
≥75	468 (18.2)	804 (26.0)	
Body mass index (kg/m^2^)	24.3 ± 3.1	24.4 ± 3.4	0.08
Underweight	54 (2.1)	97 (3.1)	0.012
Normal	1534 (59.6)	1755 (56.8)	
Overweight and obese	984 (38.3)	1237 (40.0)	
Income			0.11
Low	1183 (46.0)	1511 (49.0)	
Middle-low	689 (26.8)	788 (25.5)	
Middle-high	370 (14.4)	440 (14.2)	
High	330 (12.8)	350 (11.3)	
Education level			0.063
≤Middle school	2212 (86.0)	2720 (88.1)	
High school	278 (10.8)	279 (9.0)	
≥College	82 (3.2)	90 (2.9)	
Occupation			<0.001
White color	190 (7.4)	206 (6.7)	
Blue color	786 (30.6)	746 (24.2)	
Non-worker	1596 (62.0)	2137 (69.1)	
Residential area			0.267
Urban	2073 (80.6)	2456 (79.5)	
Rural	499 (19.4)	633 (20.5)	
National Basic Living Security			
Yes	268 (10.4)	334 (10.8)	0.664
No	2304 (89.6)	2755 (89.2)	
Current spouse status			
Yes	2022 (78.6)	2073 (67.1)	0.009
No	550 (21.4)	1016 (32.9)	

**Table 2:** Health status according to physical activity.

Variables	Active Group	Inactive Group	*p*-Value
	(*n* = 2572)	(*n* = 3089)	
Subjective health status			<0.001
Very good	85 (3.3)	60 (1.9)	
Good	647 (25.2)	584 (18.9)	
So-so	869 (33.8)	1216 (39.4)	
Bad	752 (29.2)	891 (28.8)	
Very bad	219 (8.5)	338 (11.0)	
Subjective body image			<0.001
Very thin	154 (6.0)	287 (9.3)	
Mildly thin	336 (13.1)	396 (12.8)	
Normal	1123 (43.7)	1271 (41.1)	
Chubby	777 (30.2)	902 (29.3)	
Very fatty	182 (7.1)	233 (7.5)	
Disease history			0.037
No	437 (17.0)	461 (14.9)	
Yes	2135 (83.0)	2628 (85.1)	
Number of diseases	1.62 ± 1.06	1.55 ± 1.05	0.007
0	437 (17.0)	461 (14.9)	0.054
1	834 (32.4)	996 (32.3)	
2	838 (32.6)	1005 (32.5)	
≥3	463 (18.0)	627 (20.3)	
Disease Type			
Cardiovascular	1464 (56.9)	1832 (59.3)	0.074
Respiratory	240 (9.3)	290 (9.4)	0.978
Endocrine	867 (33.7)	1103 (35.7)	0.123
Cancer	107 (4.2)	167 (5.4)	0.035
Muscular skeletal	1078 (41.9)	1330 (43.1)	0.401
Depression	191 (7.4)	238 (7.7)	0.731
Kidney	15 (0.6)	29 (0.9)	0.172
Hepatitis	33 (1.3)	44 (1.4)	0.732

**Table 3:** Lifestyle and quality of life according to physical activity.

Variables	Active Group	Inactive Group	*p*-Value
	(*n* = 2572)	(*n* = 3089)	
Sleeping time	6.4 ± 1.6	6.3 ± 1.7	0.146
≥8 h per day	1942 (75.5)	2322 (75.2)	0.794
<8 h per day	630 (24.5)	767 (24.8)	
Stress perception			0.006
Too much	116 (4.5)	185 (6.0)	
A lot	530 (20.6)	712 (23.1)	
A little	1259 (49.0)	1428 (46.2)	
Almost never	667 (25.9)	764 (24.7)	
Experiences of Depression			<0.001
Yes	416 (16.2)	663 (21.5)	
No	2156 (83.8)	2426 (78.5)	
Suicidal ideation			<0.001
Yes	461 (17.9)	756 (24.5)	
No	2111 (82.1)	2333 (75.5)	
Current smoking status			0.782
No	2484 (96.6)	2978 (96.4)	
Yes	88 (3.4)	111 (3.6)	
Skipping meals			
Breakfast	374 (14.5)	613 (19.8)	<0.001
Lunch	391 (15.2)	653 (21.1)	<0.001
Dinner	370 (14.4)	628 (20.3)	<0.001
Quality of life	0.88 ± 0.37	0.84 ± 0.19	0.036
Mobility			<0.001
Serious problem	46 (1.8)	136 (4.4)	
Some problem	911 (35.4)	442 (43.3)	
No problem	1615 (62.8)	1616 (52.3)	
Self-care			0.353
Serious problem	232 (0.9)	43 (1.4)	
Some problem	342 (13.3)	442 (14.3)	
No problem	2998 (85.8)	2604 (84.3)	
Usual activities			0.012
Serious problem	98 (3.8)	127 (4.1)	
Some problem	484 (18.8)	815 (26.4)	
No problem	1990 (77.4)	2147 (69.5)	
Pain or discomfort			0.049
Serious problem	178 (6.9)	195 (6.3)	
Some problem	779 (30.3)	1223 (39.6)	
No problem	1564 (62.8)	1671 (54.1)	
Anxiety or Depression			0.518
Serious problem	36 (1.4)	40 (1.3)	
Some problem	386 (15.0)	516 (16.7)	
No problem	2150 (83.6)	2533 (82.0)	

**Table 4:** Nutrient intakes according to physical activity.

Variables	Active Group	Inactive Group	*p*-Value
	(*n* = 2572)	(*n* = 3089)	
Energy (kcal)	1587.03 ± 516.37	1565.20 ± 501.93	0.108
Protein (g)	51.78 ± 24.22	50.72 ± 23.73	0.101
Fat (g)	22.04 ± 18.00	21.25 ± 16.23	0.084
Carbohydrate (g)	298.69 ± 99.39	294.61 ± 95.93	0.117
Fiber (g)	7.43 ± 6.13	6.89 ± 5.49	<0.001
Calcium (mg)	431.32 ± 337.53	401.55 ± 368.00	0.002
Phosphorous (mg)	915.39 ± 368.98	954.09 ± 396.73	<0.001
Iron (mg)	13.90 ± 11.05	13.65 ± 15.22	0.478
Sodium (mg)	3592.44 ± 2379.65	3753.77 ± 2595.78	0.016
Potassium (mg)	2686.77 ± 1720.67	2503.46 ± 1395.28	<0.001
Vitamin A (μgRE)	715.97 ± 1567.49	624.76 ± 859.31	0.008
Retinol (μg)	59.37 ± 266.33	50.45 ± 302.54	0.238
Carotene (μg)	3898.29 ± 9223.42	3377.29 ± 4691.26	0.009
Vitamin B_1_ (mg)	1.09 ± 0.63	1.10 ± 0.62	0.939
Vitamin B_2_ (mg)	0.88 ± 0.59	0.84 ± 0.53	0.004
Niacin (mgNE)	11.93 ± 5.91	12.40 ± 6.31	0.004
Vitamin C (mg)	99.05 ± 110.85	88.93 ± 84.79	<0.001

**Table 5:** Dietary quality according to physical activity.

Variables	Active Group	Inactive Group	*p*-Value
	(*n* = 2572)	(*n* = 3089)	
NAR			
Protein	1.16 ± 0.55	1.14 ± 0.54	0.075
Calcium	0.62 ± 0.48	0.58 ± 0.55	0.006
Phosphorous	1.31 ± 0.54	1.37 ± 0.58	0.001
Iron	1.77 ± 1.39	1.76 ± 2.00	0.814
Sodium	2.97 ± 1.97	3.03 ± 2.17	0.338
Potassium	0.77 ± 0.51	0.72 ± 0.41	<0.001
Vitamin A	1.21 ± 2.86	1.06 ± 1.49	0.034
Vitamin B_1_	1.08 ± 0.63	1.07 ± 0.61	0.658
Niacin	0.86 ± 0.43	0.90 ± 0.46	0.002
Vitamin C	1.00 ± 1.14	0.90 ± 0.87	0.002
MAR	1.10 ± 0.74	1.04 ± 0.58	0.009

**Table 6:** Linear logistic regression analyses of factors associated with quality of life (QoL) in Korean older women.

Variables		Multiple Linear Regression Model Analyses	*p*-Value
		B (95% CI)	
Physical activity		0.010 (0.001, 0.018)	0.021
Diet quality (MAR)		0.015 (0.008, 0.021)	<0.001
Income	High	0.026 (0.012, 0.040)	<0.001
	Middle-high	0.024 (0.012, 0.037)	<0.001
	Middle-low	0.023 (0.012, 0.033)	<0.001
	Low	Ref	
Education level	≥College	0.039 (0.015, 0.063)	0.001
	High school	0.027 (0.013, 0.041)	<0.001
	≤Middle school	Ref	
Subjective health status	Very good	0.018 (−0.009, 0.045)	0.188
	Good	0.019 (0.008, 0.030)	0.001
	So-so	Ref	
	Bad	−0.098 (−0.109, −0.088)	<0.001
	Very bad	−0.214 (−0.229, −0.199)	<0.001
Obesity status	Underweight	0.001 (−0.025, 0.027)	0.96
	Normal	Ref	
	Obese	−0.021 (−0.029, −0.012)	<0.001
Number of diseases		−0.015 (−0.019, −0.011)	<0.001
Without spouse		−0.033 (−0.042, −0.024)	<0.001
Stress	Almost never	Ref	
	A little	−0.011 (−0.021, −0.001)	0.035
	A lot	−0.035 (−0.048, −0.023)	<0.001
	Too much	−0.067 (−0.087, −0.047)	<0.001

CI, confidence interval; ref, reference. *p*-values were obtained from linear logistic regression.
